# COVID-19 Impact on Residential Preferences in the Early-Stage Outbreak in South Korea

**DOI:** 10.3390/ijerph182111207

**Published:** 2021-10-25

**Authors:** Bumjoon Kang, Jaewoong Won, Eun Jung Kim

**Affiliations:** 1 College of Architecture, Myongji University, Yongin 17058, Korea; bumjoon@mju.ac.kr; 2 Department of Real Estate, Graduate School of Tourism, Kyung Hee University, Seoul 02447, Korea; jwon@khu.ac.kr; 3 Department of Smart City Planning and Real Estate, Kyung Hee University, Seoul 02447, Korea; 4 Department of Urban Planning, Keimyung University, Daegu 42601, Korea

**Keywords:** post-COVID-19, residential choices, pandemic, contagious disease, urban health

## Abstract

In the early stage of the COVID-19 pandemic in South Korea, public fear or social scaring of urban living was observed, which caused people to change their daily routines. This study examines how the COVID-19 pandemic affected residential choice and perceptions of urban living. We analyzed self-reported survey data collected from 2000 participants in Seoul, Daegu, and Kyeongbuk in South Korea between 3–6 August 2020, targeting the relatively controlled period after the first COVID-19 outbreak. Logistic regression models were used to examine concerns of urban living and residence relocation consideration. Those who were aged 30 or older, regularly commuting, not feeling healthy, with a household size of two, and living in a low-rise condominium were more likely to be concerned with urban living. Those who were aged 40 or older and living in a townhouse or a single-detached house were more likely to consider moving to a less dense area. People perceived that their daily routine changed substantially after the pandemic. Certain participant groups showed concerns of urban living and relocation consideration, suggesting housing policy implications.

## 1. Introduction

In November 2019, the first reported case of the coronavirus (SAR-Cov-2) was confirmed. The World Health Organization (WHO) declared a pandemic on 11 March 2020, and it is recorded to have been over 151 million confirmed cases and over 3 million deaths as of 3 May 2021 [1]. Countries around the world have increasingly implemented regulatory actions including social distancing, indoor dining bans, and various levels of lockdowns, which affected people’s mobility and daily routines, as well as perceptions of urban living [2].

In the early stage of the pandemic, public fear or social scaring of the pandemic, coupled with governments’ preventive regulatory actions, had caused people to change their daily routines (e.g., shopping inside a store, avoiding crowds, and working/studying remotely) [2]. Rapid and comprehensive changes due to the novel, severe pandemic have been issued in a fundamental urban planning and policy agenda relating to environmental quality changes, disproportionate economic impacts and social inequalities, techno-driven approaches’ privacy concerns, absence of proactive emergency governance, risk of public transit, and lack of appropriate green and open spaces under social distancing [3]. It is obvious that the pandemic will have an irreversible impact on urban planning. However, we do not have robust consensus on how the pandemic will change our living conditions at many aspects. It is timely and important to discuss how the COVID-19 pandemic will affect residential choice, perceptions, and eventually urban changes.

### 1.1. COVID-10, Urban Density, and Planning Issues

It is often believed that high urban density may increase the risk of coronavirus infection because a higher density has been associated with a higher likelihood of contact, exposure, and interaction between people [4]. In the early stage of the U.S. pandemic, New York City had the absolute majority of COVID-19 cases and the pandemic was then regarded as a problem generally spread out in big cities. However, the pandemic is not simply due to urban density when considering cases per capita instead of absolute case numbers [5]. One study pointed out that some initial blame on density could be laid on historic negative perceptions of urban density [6].

There seems to be no consensus on the issue of urban density and COVID-19 outcomes [7,8,9,10]. Unlike the common assumption, one study argued that urban density does not necessarily increase the spread of coronavirus, explaining that, for example, Singapore and Hong Kong are denser cities than New York City and London, but they rather effectively controlled the pandemic [11,12]. Another study found that there were no significant associations between urban density and COVID-19 fatalities at the county level in the U.S. [8]. For the first-hit large cities, early studies found that connectivity was more significant than density in spreading the virus [13]. A study conducted in the Netherlands in the early stage of the pandemic also confirmed no significant associations between population density and infections, hospitalization, or mortality related to COVID-19 [14].

Conversely, there are different findings. A study found significant associations of urban density with COVID-19 cases and mortality in the U.S., and denser areas were more likely to relate to early outbreak [7]. Another study conducted in the U.S. between February and April in 2020 found that a lower population density was associated with a decrease in the instantaneous reproduction number among 211 counties [15]. A study examining South Korean cities found that urban density was significantly associated with the COVID-19 infection [16].

These two contradictory findings on the relations between COVID-19 and urban density may be both true. The “pull of the suburbs” and “push back to urban centers” may occur at the same time [17]. The former phenomena may be more relevant for the older groups and the latter for the educated younger people. Ecological fallacy or causal complications make it difficult to understand the relationships between urban density and COVID-19 [18]. For example, dense urban areas may force people to be exposed to higher infection risks while they tend to have more accessible medical resources than rural areas. In urban environments, practicing social distancing may be easy and feasible with a well-maintained infrastructure (e.g., online shopping and remote work). These factors may offset the high density of large cities’ vulnerability to the coronavirus [9].

Urban density and the pandemic issue evolved into discussions on spatial structure and urban policy as the pandemic continued to spread globally. One study argued that the pandemic may force some ‘urban exodus’ phenomena at the microgeographic scale, however, the ‘urban buzz’ will be continued or could even be accelerated after the pandemic [13]. Conversely, a study argued that the general bid–rent curve will be flattened in urban areas, suggesting land use distribution might be reshaped after the pandemic [19]. There is an argument that land use configuration needs to be changed due to preferred access to green infrastructure in a post COVID-19 world [20]. At this point, many policy claims are based on assumptions and arguments. However, scientific studies based on empirical data are few and still inconclusive. We need empirical data in diverse urban contexts to guide more informed urban policy development.

The spread of the pandemic differed by circumstance of cities and countries. There are large differences in urban contexts, public health systems, natural environments, and governmental policy settings [21]. Therefore, the pandemic control strategies and the pandemic’s health, economy, and social impacts were incomparable across geography and time. For example, neighborhood-level wealth was negatively associated with the COVID-19 incidence in New York City in the U.S. at the first wave of the pandemic, but it was not in London in the U.K. or in Madrid in Spain in the same time frame [13]. A study from London in the U.K. focusing on the very early stage (March and April, 2020) found that the higher COVID-19 fatality rate was associated with higher neighborhood-level deprivation, pointing out low health care accessibility of poor neighborhoods [22]. Another study examining Hong Kong and South Korea found no associations between neighborhood-level wealth and COVID-19 infection [23]. One study discussed that the geographic and behavioral scales may complicate environmental correlates of COVID-19 outcomes [24]. To have a better understanding of the highly context-specific pandemic phenomena, we need to have data focusing on specific contexts, which requires an in-depth study on individual, unique cases of pandemic situations.

**Figure 1:**
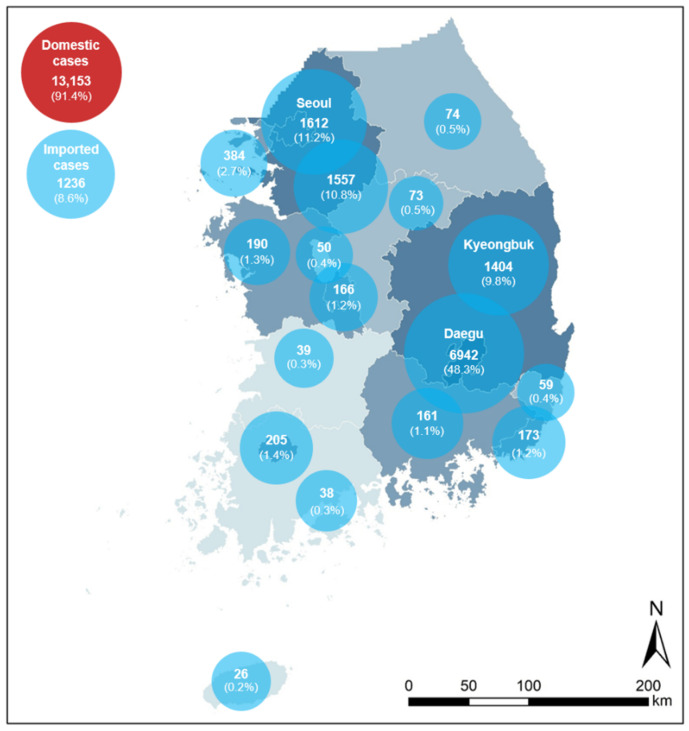
Map of the study areas and confirmed COVID-19 cases as of 3 August 2020.

Our study timeframe is August of 2020. It was about 3–4 months after South Korea experienced the first wave of the COVID-19 outbreak, and before the second wave starting from the middle of August. The first wave was a local pandemic severely hitting the City of Daegu (hereafter, Daegu), the third biggest city in South Korea, and affecting the city’s surrounding rural areas in the province of Gyeongsangbuk-do (known as Kyeongbuk). During the first wave, the City of Seoul (hereafter, Seoul), the capital and largest city of South Korea, about 240 km away from Daegu, was not seriously hit by the pandemic, but it became the most hit area in the coming second wave as a national pandemic. We selected these three regions, Seoul, Daegu, and Kyeongbuk (Figure 1), as the study area and set the study timeframe between the first and the second waves, when a local outbreak was developing into a national wide pandemic. This study aims to record people’s perception (public scaring of urban living) after the COVID-19 outbreak, and to examine whether the pandemic affected people’s public scaring and perceptions of urban living in the study areas covering two major cities and one suburban-rural area that were most affected by the pandemic in the country.

### 1.2. Pandemic in the Study Area

In South Korea, as of 14 July 2021, 171,911 confirmed cases were identified and the fatality rate was 1.2% (incidence of 332 per 100,000 population) [25]. From the beginning of the pandemic, South Korean governments have established the 3T Strategy: Testing, Tracing, and Treatment. In brief, the strategy employed widespread testing to identify carriers at the earliest possible time, prompt tracing to prevent secondary infections, and transferring confirmed patients to medical institutions [26]. The 3T Strategy was considered relatively successful in containing virus infections while minimizing economic and social impacts [27]. However, South Korea was one of the first hit countries even before the World Health Organization declared the COVID-19 outbreak a global pandemic on 11 March 2020 [28] and had experienced severe local outbreak surges in the early stage before many other countries.

**Figure 2:**
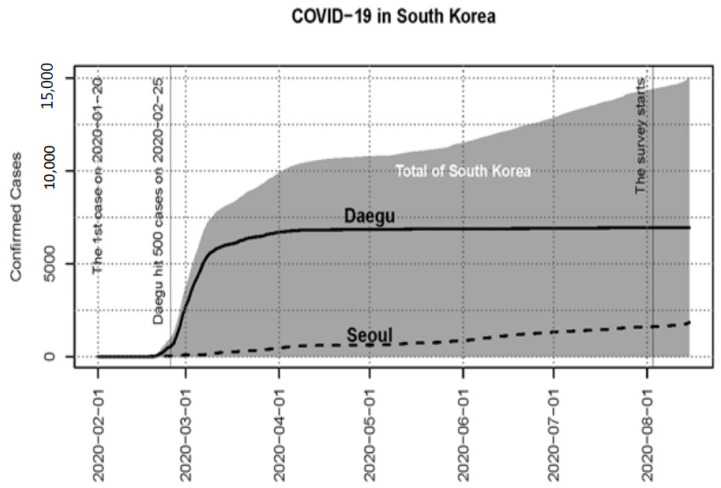
COVID-19 accumulated confirmed cases in South Korea in the context of the survey time point.

In South Korea, the first case of COVID-19 was confirmed on 20 January 2020. Shortly after the first confirmed case, South Korea had the first large outbreak outside of China, which was centered in Daegu. By 29 February 2020, Daegu reported more than 2000 confirmed cases by February 29 and over 5000 by March 7. At that point, 75.1% out of the national total of 6767 confirmed cases were from Daegu and Kyeongbuk [25]. This outbreak was considered as the first wave outbreak in South Korea. After the first wave of the Daegu surge, South Korea had a relatively controlled period between April and July, having daily new confirmed cases less than 100, except for one day, with an accumulated total of 14,305 by 31 July 2020 (Figure 2). In the meantime, many other countries including Italy, Spain, Iran, and the U.S. experienced overwhelming COVID-19 outbreaks. For example, the U.S. had more than 4.6 M confirmed cases by the end of July. Several countries’ outbreak triggered global public fear and anxiety of urban living.

Shortly after, South Korea faced the second wave with the daily peak of 441 new cases on 27 August 2020. Public distress levels were high. According to Pew Research Center’s survey reported on July 2020, about 22% of U.S. adults moved or know someone who moved to reduce their risk of contracting the COVID-19 virus [29]. One study that conducted survey data covering our study areas (Seoul and Daegu) found that general perceived stress levels and stress related to COVID-19 were significantly associated with the local prevalence of COVID-19 [30]. It was certain that public fear and scaring affected people’s perceptions of urban living as the global pandemic progressed.

This was a unique time point. South Korea was the first country that experienced the unprecedented COVID-19 outbreak and observed the global pandemic in other countries. Because it was obvious that the second or third waves would come back to South Korea, public fear of the virus may have shaped people’s attitude or perceptions. South Koreans were exposed to media delivering shocking pandemic news from other countries. This study attempts to capture the perception changes that possibly occurred during the time frame to find urban planning implications of the pandemic by collecting survey data from areas with variable COVID-19 prevalence levels.

## 2. Materials and Methods

### 2.1. Data

To examine people’s perceptions related to COVID-19, we collected and analyzed survey data specifically designed for this study. An online stated preference survey (SPS) was conducted during 3–6 August 2020 by a research company specializing in online research and panels. The study period, as explained above, was set to capture people’s perceptions when the COVID-19 outbreak was managed and controlled after the first outbreak. We selected the survey company because it had records of on-going contracts of online surveys on the behalf of the Seoul Metropolitan Government where we planned to collect a majority of the data. The study area covered Daegu and Kyeongbuk, where the first COVID-19 outbreak started, and Seoul, which has the most economic activities and the greatest geographic connections in South Korea as the capital city. Details of the three areas are explained in Table 1.

Valid surveys came from participants aged 18 years or older living in either Seoul, Daegu, or Kyeongbuk. Using the company’s online panel sampling frame, which stratifies sex, age, and geography, we aimed to have a sampling error of ±2.5% in Seoul and ±4.5% in Daegu and Kyeongbuk at the 95% significance level. Because Daegu and Kyeongbuk share their boundaries, we used the same sampling frame for the two areas. We aimed to collect 2000 valid responses (1500 from Seoul and 500 from Daegu and Kyeongbuk). The sample size was chosen to get the sampling error explained above. For Daegu and Kyeongbuk, we had to accept a large error due to the small paneling pool.

The SPS was designed to ask participants to report their socio-demographics and perceived changes in their daily routines after the COVID-19 outbreak. Perceptions may be affected by one’s personal COVID-19 experiences [30], and therefore, we included a variable if they, their family members, or acquaintances, experienced a COVID-19 test, quarantine, or diagnosis. COVID-19 was about seven months progressed since the confirmed case was reported and 3–4 months after the Daegu surge so that we might have robust results on people’s changed daily routines and perceptions. The SPS was expected to be completed within 20 min.

Figure 2 shows the COVID-19 pandemic trends across the timeline and our study time frame (between 3–6 August 2020). The study was approved by the research team’s Institutional Review Board. The initial data were collected and pre-processed by SPSS 26.0 (IBM Corp., Armonk, NY, USA).

### 2.2. Analysis

In this study, we examined factors affecting changes in perception of urban living after experiencing the pandemic. Selected key outcome variables capturing the changes of perceptions are (i) becoming concerned about living in a big city (city concern) and (ii) starting to consider moving to a suburban or less urban area (moving out of the city). Other than the outcome variables, we collected information showing how a participant’s daily routines changed (e.g., staying at home longer than before the pandemic, spending less time doing physical activity than before, and thinking one’s daily routines changed in general after the pandemic).

With the variables, we conduct three analyses. First, we examined the key outcome variables by separating the study sample into three groups (Seoul, Daegu, and Kyeongbuk). Chi-square tests were used to examine if the three groups had different perception changes. Second, we focused on the key outcome variables and checked if personal, home-related, COVID-19 affected, and COVID-19-related daily routine changes were associated with “city concern” or “moving out of the city” with chi-square tests. Finally, we established logistic regressions to explain whether “city concern” and “moving out of the city” were associated with predictor variables. In these models, the perception variables showing COVID-19 daily routine changes were excluded if they had statistically significant dependencies with the key outcome variables to avoid the variables’ potential autocorrelations with other predictors. All analyses were conducted with R 4.03 (R Core Team, Vienna, Austria).

## 3. Results

A total of 15,651 participants were invited via email containing the structured online survey form. About 22% (3462) replied that they received the invitation. We collected survey responses until the final sample reached to 2000 valid responses, including 1500 from Seoul and 500 from Daegu and Kyeongbuk (245 from Daegu and 255 from Kyeongbuk). The final response rate was 12.8% without significant differences between two spatial sampling frameworks (13.0% in Seoul vs. 12.2% in Daegu and Kyeongbuk).

Table 2 shows the sample descriptive statistics. All three samples had nearly equal distribution of the sex variable and covered various age and income groups, while the Seoul sample was younger and richer, more likely to be employed (home makers were coded as employed), perceived healthier, and had a lower proportion of 1- or 2-person households than the Daegu and the Kyeongbuk samples. Across the three samples, the majority were living in high-rise condominiums, which is the most common housing type in South Korea in both urban areas.

Table 3 shows how participants perceived that their daily routines were influenced by COVID-19. Differences across the sample groups were examined using chi-square tests. Among those who regularly commute, about half of the Seoul and Daegu participants and 36% of Kyeongbuk participants experienced working or studying remotely from home. Due to the first outbreak from Daegu, about 7% of the participants experienced COVID-19 (test, quarantine, or diagnosis of oneself or close acquaintance), while about 4% of Seoul and Kyeongbuk participants experienced COVID-19. Among all the samples, daily life routines were changed after the pandemic. For example, nearly 70% of all samples reported that their time staying at home increased. About 39–45% from the three areas said their time spent in physical activity decreased. In general, most participants agreed that their daily routines had changed after the pandemic (72.7–76.3%). Among those who agreed, about half (45.5–51.3%) answered that their changed daily routines would not reverse even after the pandemic was over. Thus, about 33–39% of the entire sample perceived that their daily routine may not come back after the pandemic.

About 32% from Seoul, 25% from Daegu, and 19% from Kyeongbuk agreed that they became concerned with living in a city after the pandemic, which is the only statistically different post-COVID-19 perception change across the study area (*p* < 0.001). Similarly, the Seoul sample was more likely to consider moving to a suburban or less urban area than the other samples (23% vs. 18% or 19%). However, the difference did not meet the statistical significance threshold.

Regarding the outcome variables (“city concern” and “moving out of the city”), many factors appeared statistically significant as shown in Table 4. Interestingly, three variables capturing daily routine changes (staying at home longer than before, having less physical activity, and daily routine change in general) were all dependent on the outcome variables at the significant level of *p* < 0.001.

Table 5 shows the logistic regression model coefficients on “city concern” and “moving out of the city”. Those who were aged 30 or older, commuting, not feeling healthy, with a household size of two, living in a low-rise condominium, staying at home longer than before, and living in Seoul were more likely to be concerned with urban living (Model 1). Those who were aged 40 or older, living in a townhouse or a single-detached house, and staying home longer than before were more likely to consider moving to a less dense area (Model 2). In both models, the COVID-19 experience was not significant.

## 4. Discussion

This study, focusing on a unique time point of the COVID-19 outbreak in South Korea, revealed that COVID-19 had substantially changed people’s daily routines and perceptions of urban living in the study area. Daegu was the first severe epidemic starting point. Kyeongbuk, immediately adjacent to Daegu, was directly affected by the Daegu epidemic. Conversely, Seoul was not severely hit at the point of the survey, but substantial perception changes were not significantly different across areas, except for remote working experience and “city concern.” It is interesting that about half of the participants agreed that their daily routines changed after the pandemic. Even after the pandemic is over, their daily routines will not come back as before (33–39% of the entire sample). They experienced that a longer stay time at home reduced time spent in physical activity. They perceived their increased concern of urban living and were considering moving to low-density areas. These changes, especially for 33–39% of the sample, are not likely to be reversed after the pandemic is over. It is difficult to predict the post-COVID-19 world at this point [13]. However, our findings suggest important implications for future urban planning as follows.

First, if the changes continue, housing markets may be segmented and differentiated by age group. Older populations were more likely concerned with urban living and seemed to consider moving to less urbanized areas than the youngest age group (between 20 and 29 years old). More settled populations may seek safety over “urban buzz” while younger populations may need urban amenities [13]. It is possible that the youngest age group believed that they were relatively safer from the virus and more resilient to infections than older populations. Alternatively, the youngest age group may have substantially different residential preferences than older groups, regardless of the pandemic situations. Even before the pandemic, the millennial generation strongly preferred cities over suburbs. Additionally, there were generational residential patterns. The younger generation’s urban preference resulted from economic and demographic factors [31,32]. Thus, we need more studies to disentangle the generational phenomena. However, our data clearly indicate that the pandemic may have minimal effects on younger generation’s housing location choices in South Korea.

Second, public transit agencies may prepare for post-COVID-19 single occupancy vehicle (SOV) demands. Commuters were more likely to be concerned with urban living than non-commuters. However, commuters were not considering the actual moving out of cities. In South Korea, major employment centers are located in city centers. Commuters’ concern or fear of the virus may be true. However, these may not be sufficient factors to change commuters’ residential choice. After many global pandemics from contagious diseases such as SARS or swine flu, it was consistently found that people showed precautionary behaviors such as avoiding public transit use [8]. If the commuters’ concern diverts their mode changes of transportation from public transit to SOV, which is considered safe from infectious disease, it is possible that SOV demands may surge after the economy comes back. As of 2012 in Seoul, the share of public transportation was 74% out of all transportation modes [33]. A small shift from this to SOV could be an immense issue after the pandemic is over. In the U.S., transit ridership sharply declined after the pandemic started due to transit users fear and transit authorities’ reduced operation policies [34].

Third, future housing buyers may prefer housing complexes isolated from the public. In our study, those living in a high-rise condominium did not have concerns with urban living nor considered moving out of the city. Conversely, those living in a low-rise condominium had an increased concern of urban living. Those living in a townhouse or a single-detached house considered moving out of the city. While apartment or town house residents in Brazil significantly reduced their physical activity time and traveling time during the pandemic, possibly due to the fear of public exposure [35], the residents’ behavioral preferences might depend on urban development contexts. In South Korea, high-rise condominiums are typically located in high-density “planned” housing complexes, while the others are in low-density “unplanned”, organic-form neighborhoods. People living in a planned housing complex may feel safe because it typically has private gates with entrance control, although it has relatively high residential density. Low-rise condominiums and single detached houses are usually open to the public, which may increase residents’ fear of uncontrolled contacts with unknowns. In the exploratory stage of the research project, the research team conducted unstructured interviews with a small group of participants directly affected by COVID-19. One of the interviewees stated that she felt her house is safe because her housing unit is a part of a well-managed, planned, large high-rise condominium complex. It seems that controlled environments were more important than density itself. A larger, city-level analysis in South Korea found that spatial connectivity had bigger impacts on proliferating the COVID-19 pandemic than urban density [16]. Thus, people may seek controlled neighborhoods. This could be a future planning issue if future housing buyers want gated communities with no or limited public access.

Fourth, increased time spent at home may reduce urban living preferences. An increase in time staying at home can reduce the need to live in the city center and may consequently lead to considerations of moving out of the city. Over the past few decades, increasing evidence suggest that information and communication technology (ICT) can play a role in decentralization or centralization in urban spatial structure [36]. The results of this study, which show that the time spent at home increased due to the pandemic, may further accelerate decentralization in the era of high-quality ICT. High quality ICT such as high-speed internet and smart technologies may replace conventional face-to-face daily routines, which may reduce the attractiveness of living in a city center.

Fifth, unexpectedly, the COVID-19 experience was not significant in both models in Table 5. This means that personal exposure to the disease did not cause the perception changes. Public fear of the pandemic may change people’s perception of urban living. Our study was conducted when the pandemic was relatively well-managed in early August, 3–4 months after the first outbreak. If such fear fades out in some future time, it is possible that the changed perception may be reversed. As we have been facing the third and fourth waves of the pandemic at the point of this manuscript preparation, people’s fear and public scaring may have evolved into different stages. Our aim was to record people’s perception changes at a certain point of time in significant locations. This study focused on a special case of South Korea as the pandemic progressed into a national scale.

Finally, in Model 1 (Table 5), those living in Kyeongbuk were more likely concerned with living in a city than those in Seoul. Considering that Kyeongbuk is much less dense than Seoul, Kyeongbuk residents’ negative perception toward city living may be associated with the fact that the very first local surge occurred in Kyeongbuk area. Unprecedented pandemic may elevate people’s fear, public scaring, and may affect residential preferences, although it is not clear whether the effect is temporary or not.

This study is subject to several limitations. This study is based on one-time, stated preference data. Follow-up observations and different measurement strategies may be needed to verify our findings. The purpose of our study is to present one observation to start discussions on the topic of the pandemic and urban policy directions. Second, the sample used in this study was representative of the regional populations with respect to age and sex. Due to the incomparable variable structure, other variables were not used to test differences between the sample and the population. Cautions are needed when interpreting our findings. Third, this study was conducted as a snapshot of the early stages of the COVID-19 pandemic. In the unprecedentedly long-running pandemic, people’s fear of COVID-19 and demands of urban space in their daily lives must have changed. Finally, detailed demographic characteristics may be useful to disentangle correlated explanatory variables or proxies and for international comparison studies.

## 5. Conclusions

In the early stage of the COVID-19 pandemic in South Korea, people perceived that their time staying at home significantly increased and time spent in physical activity significantly decreased, and generally agreed that daily routines changed substantially. Our findings on changed preferences suggest that planning policy needs to consider potential housing market segregation by age group, decreased demand of public transit, expected housing complexes’ building type change with high-level entrance control, and people’s changed daily routines with increased time spent at home.

## Figures and Tables

**Table 1:** Conditions of the study area as of 3 August 2020 (start of the study period).

Area	Population *N* (%)	Population Density (Person/km^2^)	COVID-19		
			Confirmed Cases	Deaths	Incidents
			*N* (%)	*N* (%)	per 100,000
South Korea	51,839,852 (100.0)	516.3	14,389 (100.0)	301 (100.0)	27.8
Seoul	9,715,429 (18.7)	16,053.3	1612 (16.2)	11 (4.3)	16.6
Daegu	2,428,022 (4.7)	2748.2	6942 (69.7)	191 (74.6)	285.9
Kyeongbuk	2,644,001 (5.1)	138.9	1404 (14.1)	54 (21.1)	53.1

Sources: Korean Statistical Information Service (population as of July 2020; administration area as of 2019); Korea Disease Control and Prevention Agency (COVID-19 statistics as of 3 August 2020).

**Table 2:** Sample descriptive statistics.

Socio-Demographic Variables		Seoul (*N* = 1500)		Daegu (*N* = 245)		Kyeongbuk (*N* = 255)	
		Count	(%)	Count	(%)	Count	(%)
Sex	Male	736	(49.1)	122	(49.8)	134	(52.5)
	Female	764	(50.9)	123	(50.2)	121	(47.5)
Age group	29 or younger	340	(22.7)	51	(20.8)	46	(18.0)
	30–39	302	(20.1)	41	(16.7)	41	(16.1)
	40–49	309	(20.6)	53	(21.6)	52	(20.4)
	50–59	306	(20.4)	58	(23.7)	62	(24.3)
	60 or older	243	(16.2)	42	(17.1)	54	(21.2)
Household income KRW 1,000,000/month USD 1 ≈ KRW 1194 as of 1 August 2020	<100	41	(2.7)	18	(7.3)	9	(3.5)
	100–200	113	(7.5)	28	(11.4)	38	(14.9)
	200–300	232	(15.5)	37	(15.1)	59	(23.1)
	300–400	233	(15.5)	46	(18.8)	40	(15.7)
	400–500	290	(19.3)	41	(16.7)	47	(18.4)
	≥500	591	(39.4)	75	(30.6)	62	(24.3)
Employment status	Not employed	127	(8.5)	33	(13.5)	36	(14.1)
	Employed	1224	(81.6)	187	(76.3)	195	(76.5)
	Students	149	(9.9)	25	(10.2)	24	(9.4)
Commute	Yes	1001	(66.7)	156	(63.7)	153	(60.0)
	No	499	(33.3)	89	(36.3)	102	(40.0)
Feel healthy	Agree	691	(46.1)	104	(42.4)	92	(36.1)
	Neither agree or disagree	613	(40.9)	110	(44.9)	137	(53.7)
	Disagree	196	(13.1)	31	(12.7)	26	(10.2)
**House-Related Variables**							
Household size	1	184	(12.3)	23	(9.0)	23	(31.4)
	2	298	(19.9)	62	(24.3)	62	(26.7)
	3	432	(28.8)	80	(31.4)	80	(8.6)
	4	471	(31.4)	68	(26.7)	68	(26.7)
	5+	115	(7.7)	22	(8.6)	22	(8.6)
Residential location	CBD	80	(4.9)				
	North-East	464	(28.4)				
	North-West	316	(19.3)				
	South-East	316	(19.3)				
	South-West	458	(28.0)				
Housing type	High-rise condominium	827	(55.1)	156	(63.7)	170	(66.7)
	Low-rise condominium	165	(11.0)	19	(7.8)	12	(4.7)
	Town house	305	(20.3)	23	(9.4)	10	(3.9)
	Single detached	124	(8.3)	35	(14.3)	44	(17.3)
	Dormitory	65	(4.3)	9	(3.7)	15	(5.9)
	Others	14	(0.9)	3	(1.2)	4	(1.6)

**Table 3:** Post-COVID-19 changes.

Changes after the COVID-19 Outbreak		Seoul		Daegu		Kyeongbuk		*p* *
		Count	(%)	Count	(%)	Count	(%)	
Start remote work/study	Yes	406	(49.0)	64	(48.1)	43	(36.1)	0.031
	No	422	(51.0)	69	(51.9)	76	(63.9)	
COVID-19 experience	Yes	60	(4.0)	17	(6.9)	9	(3.5)	0.089
	No	1440	(96.0)	228	(93.1)	246	(96.5)	
Stay at home	Increase by ≥1 h	1068	(71.2)	171	(69.8)	175	(68.6)	0.353
	Not change or change <±1 h	412	(27.5)	73	(29.8)	79	(31.0)	
	Decrease by ≥1 h	20	(1.3)	1	(0.4)	1	(0.4)	
Physical activity	Increase	202	(13.5)	30	(12.2)	34	(13.3)	0.513
	Not change significantly	635	(42.3)	106	(43.3)	122	(47.8)	
	Decrease	663	(44.2)	109	(44.5)	99	(38.8)	
Daily routine change in general	Agree	1145	(76.3)	178	(72.7)	186	(72.9)	0.445
	Neither agree nor disagree	279	(18.6)	54	(22.0)	58	(22.7)	
	Disagree	76	(5.1)	13	(5.3)	11	(4.3)	
Even after the pandemic is over, daily routine will change (Only asked among “agree” in the above)	Agree	770	(51.3)	112	(45.7)	116	(45.5)	0.333
	Neither agree nor disagree	292	(19.5)	56	(22.9)	56	(22.0)	
	Disagree	83	(5.5)	10	(4.1)	14	(5.5)	
	NA	355	(23.7)	67	(27.3)	69	(27.1)	
Concerned with living in a city	Agree	483	(32.2)	60	(24.5)	49	(19.2)	<0.001
	Neither agree nor disagree	415	(27.7)	68	(27.8)	76	(29.8)	
	Disagree	602	(40.1)	117	(47.8)	130	(51.0)	
Consider moving to a suburban or less urban area	Agree	346	(23.1)	44	(18.0)	49	(19.2)	0.219
	Neither agree nor disagree	266	(17.7)	52	(21.2)	44	(17.3)	
	Disagree	888	(59.2)	149	(60.8)	162	(63.5)	

* Chi-square test results.

**Table 4:** Chi-square test *p*-values, indicating independencies between variables.

Variables	Concerned with Living in a City	Consider Moving to a Suburban or Less Urban Area
Personal characteristics		
Sex	0.199	<0.001
Age group	<0.001	<0.001
Income	0.511	0.311
Employment status	0.014	0.003
Commuting or not	0.058	0.284
Feel healthy	<0.001	<0.001
Home-related characteristics		
Household size	0.271	0.311
Residential location	0.002	0.207
Housing type	0.392	0.009
COVID-19 affected		
Start remote work/study	0.039	0.367
COVID-19 experience	0.224	0.059
COVID-19 daily routine change		
Stay at home	<0.001	<0.001
Physical activity	<0.001	<0.001
Daily routine change	<0.001	<0.001

**Table 5:** Models explaining post-COVID-19 residential preferences.

		Model 1 Y = Concerned with Living in a City			Model 2 Y = Considering Moving to a Suburban or Less Urban Area		
Coefficients		Est.	SE	*p* *	Est.	SE	*p* *
(Intercept)		−2.29	0.26	<0.001	−2.98	0.31	<0.001
Sex	Female	−0.04	0.11	0.706	−0.14	0.12	0.246
Age	30–39	0.50	0.19	**0.009**	0.36	0.23	0.121
(ref: 20–29)	40–49	0.47	0.19	**0.015**	0.68	0.23	**0.003**
	50–59	0.39	0.19	**0.046**	0.83	0.23	**<0.001**
	60+	0.45	0.21	**0.031**	1.13	0.24	**<0.001**
Income [KRW 1M]	100–200	−0.14	0.23	0.545	0.02	0.25	0.949
(ref: <100)	200–300	−0.05	0.19	0.779	0.09	0.21	0.663
	300–400	0.29	0.17	0.083	0.07	0.18	0.710
	400–500	−0.38	0.15	**0.009**	−0.31	0.16	0.054
	≥500	0.12	0.13	0.373	0.05	0.15	0.735
Employment status	Employed	0.15	0.22	0.508	0.22	0.24	0.356
(ref: not employed)	Student	0.27	0.28	0.335	0.23	0.34	0.489
Commute	Yes	0.29	0.13	**0.027**	0.19	0.14	0.190
Feel healthy	No	0.43	0.15	**0.003**	0.13	0.16	0.434
HHD size	2	0.41	0.18	**0.020**	0.04	0.19	0.828
(ref: 1)	3	0.00	0.15	0.999	0.19	0.17	0.252
	4	−0.07	0.12	0.593	−0.15	0.13	0.253
	5+	0.08	0.10	0.451	0.06	0.11	0.569
Housing type	Low-rise condo.	0.35	0.17	**0.047**	0.31	0.20	0.113
(ref: high-rise condominium)	Town house	0.28	0.14	0.055	0.35	0.16	**0.027**
	Single detached	0.06	0.18	0.727	0.66	0.18	**<0.001**
	Dormitory	0.41	0.29	0.152	0.32	0.33	0.319
	Others	0.75	0.48	0.119	−0.12	0.65	0.851
COVID-19 experience	Yes	0.12	0.25	0.626	0.43	0.26	0.092
Stay home (ref: no change or decrease)	Increase	0.94	0.13	**<0.001**	0.93	0.14	**<0.001**
City/region	Daegu	−0.35	0.17	**0.035**	−0.34	0.19	0.066
(ref: Seoul)	Kyeongbuk	−0.66	0.18	**<0.001**	−0.22	0.18	0.215

* Significant *p*-values shown in bold.

## Data Availability

Not applicable.

## References

[1] World Health Organization WHO Coronavirus (COVID-19) Dashboard. https://covid19.who.int/.

[2] Shamshiripour A., Rahimi E., Shabanpour R., Mohammadian A.K. (2020). How is COVID-19 reshaping activity-travel behavior? Evidence from a comprehensive survey in Chicago. Transp. Res. Interdiscip. Perspect..

[3] Sharifi A., Khavarian-Garmsir A.R. (2020). The COVID-19 pandemic: Impacts on cities and major lessons for urban planning, design, and management. Sci. Total Environ..

[4] Jamshidi S., Baniasad M., Niyogi D. (2020). Global to USA County Scale Analysis of Weather, Urban Density, Mobility, Homestay, and Mask Use on COVID-19. Int. J. Environ. Res. Public Health.

[5] Loh T.H., Leinberger C. How Fear of Cities Can Blind Us from Solutions to COVID-19. https://nextcity.org/urbanist-news/entry/how-fear-of-cities-can-blind-us-from-solutions-to-covid-19.

[6] McFarlane C. (2021). Repopulating density: COVID-19 and the politics of urban value. Urban Stud..

[7] Carozzi F., Provenzano S., Roth S. (2020). Urban Density and COVID-19.

[8] Hamidi S., Sabouri S., Ewing R. (2020). Does density aggravate the COVID-19 pandemic? Early findings and lessons for planners. J. Am. Plan. Assoc..

[9] Overman H.G., Nathan M. (2021). Will coronavirus cause a big city exodus?. Cent. Mag. Econ. Perform. 601 Cent. Econ. Perform. LSE.

[10] Teller J. (2021). Urban density and COVID-19: Towards an adaptive approach. Build. Cities.

[11] Patino M. Why Asian Countries Have Succeeded in Flattening the Curve. Bloomberg CityLab 1 April 2020. https://www.bloomberg.com/news/articles/2020-03-31/how-to-make-people-stay-home.

[12] Salama A.M. After Coronavirus: How Seasonal Migration and Empty Centres might Change Our Cities. https://theconversation.com/after-coronavirus-how-seasonal-migration-and-empty-centres-might-change-our-cities-139439.

[13] Florida R., Rodríguez-Pose A., Storper M. (2021). Cities in a post-COVID world. Urban Stud..

[14] Boterman W.R. (2020). Urban-Rural Polarisation in Times of the Corona Outbreak? The Early Demographic and Geographic Patterns of the SARS-CoV-2 Epidemic in the Netherlands. Tijdschr. Econ. Soc. Geogr..

[15] Rubin D., Huang J., Fisher B.T., Gasparrini A., Tam V., Song L., Wang X., Kaufman J., Fitzpatrick K., Jain A. (2020). Association of social distancing, population density, and temperature with the instantaneous reproduction number of SARS-CoV-2 in counties across the United States. JAMA Netw. Open.

[16] Jo Y., Hong A., Sung H. (2021). Density or Connectivity: What Are the Main Causes of the Spatial Proliferation of COVID-19 in Korea?. Int. J. Environ. Res. Public Health.

[17] Florida R. The Forces That Will Reshape American Cities. https://www.bloomberg.com/news/features/2020-07-02/how-coronavirus-will-reshape-u-s-cities.

[18] Nathan M. The City and the Virus. https://maxnathan.medium.com/the-city-and-the-virus-db8f4a68e404.

[19] Nanda A., Thanos S., Valtonen E., Xu Y., Zandieh R. (2020). Forced homeward: The COVID-19 implications for housing. Town Plan. Rev..

[20] Mell I., Whitten M. (2021). Access to Nature in a Post COVID-19 World: Opportunities for Green Infrastructure Financing, Distribution and Equitability in Urban Planning. Int. J. Environ. Res. Public Health.

[21] Diao Y., Kodera S., Anzai D., Gomez-Tames J., Rashed E.A., Hirata A. (2021). Influence of population density, temperature, and absolute humidity on spread and decay durations of COVID-19: A comparative study of scenarios in China, England, Germany, and Japan. One Health.

[22] Harris R. (2020). Exploring the neighbourhood-level correlates of COVID-19 deaths in London using a difference across spatial boundaries method. Health Place.

[23] Koh K., Park S., Chan Y.Y., Cheung T.H. (2021). Does Socioeconomic Status Matter? A Study on the Spatial Patterns of COVID-19 in Hong Kong and South Korea. Coronavirus (COVID-19) Outbreaks, Environment and Human Behaviour: International Case Studies.

[24] Kar A., Motoyama Y., Carrel A.L., Miller H.J., Le H.T. (2021). COVID-19 Exacerbates Unequal Food Access. Appl. Geogr..

[25] Central Disease Control Headquarters Coronavirus Disease-19. Republic of Korea. http://ncov.mohw.go.kr/.

[26] Park Y., Huh I.S., Lee J., Kang C.R., Cho S.-I., Ham H.J., Kim H.S., Kim J.-I., Na B.J., Lee J.Y. (2020). Application of testing-tracing-treatment strategy in response to the COVID-19 outbreak in Seoul, Korea. J. Korean Med. Sci..

[27] Kim J.-H., An J.A.-R., Oh S.J., Oh J., Lee J.-K. Emerging COVID-19 Success Story: South Korea Learned the Lessons of MERS. https://ourworldindata.org/covid-exemplar-south-korea.

[28] Cucinotta D., Vanelli M. (2020). WHO declares COVID-19 a pandemic. Acta Biomed. Atenei Parm..

[29] Cohn D.V. About a Fifth of U.S. Adults Moved Due to COVID-19 or Know Someone Who Did. https://www.pewresearch.org/fact-tank/2020/07/06/about-a-fifth-of-u-s-adults-moved-due-to-covid-19-or-know-someone-who-did/.

[30] Kim M., Park I.-H., Kang Y.-S., Kim H., Jhon M., Kim J.-W., Ryu S., Lee J.-Y., Kim J.-M., Lee J. (2020). Comparison of psychosocial distress in areas with different COVID-19 prevalence in Korea. Front. Psychiatry.

[31] Myers D. (2016). Peak millennials: Three reinforcing cycles that amplify the rise and fall of urban concentration by millennials. Hous. Policy Debate.

[32] Lee H. (2020). Are millennials coming to town? Residential location choice of young adults. Urban Aff. Rev..

[33] The Seoul Institutes Modal Share. https://data.si.re.kr/node/seoulgwa-segyedaedosi/51.

[34] Liu L., Miller H.J., Scheff J. (2020). The impacts of COVID-19 pandemic on public transit demand in the United States. PLoS ONE.

[35] Browne R.A.V., Cabral L.L.P., Freire Y.A., Macêdo G.A.D., Oliveira G.T.A., Vivas A., Elsangedy H.M., Fontes E.B., Costa E.C. (2021). Housing type is associated with objectively measured changes in movement behavior during the COVID-19 pandemic in older adults with hypertension: An exploratory study. Arch. Gerontol. Geriatr..

[36] Dadashpoor H., Yousefi Z. (2018). Centralization or decentralization? A review on the effects of information and communication technology on urban spatial structure. Cities.

